# Clinical Significance of NKD Inhibitor of WNT Signaling Pathway 1 (NKD1) in Glioblastoma

**DOI:** 10.1155/2023/1184101

**Published:** 2023-03-17

**Authors:** Lijun Li, Ruiying Gao, Weizhong Huangfu, Fang Zhang, Ruixia Wang

**Affiliations:** ^1^Department of General Practice, Affiliated Hospital of Inner Mongolia Medical University, Hohhot, Inner Mongolia 010000, China; ^2^Department of Neurology Ward A, Affiliated Hospital of Inner Mongolia Medical University, Hohhot, Inner Mongolia 010000, China

## Abstract

**Introduction:**

As the most malignant type of gliomas, glioblastoma is characterized with disappointing prognosis. Here, we aimed to investigate expression and function of NKD inhibitor of Wnt signaling pathway 1 (NKD1), an antagonist of Wnt-beta-catenin signaling pathways, in glioblastoma.

**Methods:**

The mRNA level of NKD1 was firstly retrieved from TCGA glioma dataset to evaluate its correlation with clinical characteristics and its value in prognosis prediction. Then, its protein expression level in glioblastoma was tested by immunohistochemistry staining in a retrospectively cohort collected from our medical center (*n* = 66). Univariate and multivariate survival analyses were conducted to assess its effect on glioma prognosis. Two glioblastoma cell lines, U87 and U251, were used to further investigate the tumor-related role of NKD1 through overexpression strategy in combination with cell proliferation assays. Immune cell enrichment in glioblastoma and its correlation with NKD1 level was finally assessed using bioinformatics analyses.

**Results:**

NKD1 shows a lower expression level in glioblastoma compared to that in the normal brain or other glioma subtypes, which is independently correlated to a worse prognosis in both the TCGA cohort and our retrospective cohort. Overexpressing NKD1 in glioblastoma cell lines can significantly attenuate cell proliferation. In addition, expression of NKD1 in glioblastoma is negatively correlated to the T cell infiltration, indicating it may have crosstalk with the tumor immune microenvironment.

**Conclusions:**

NKD1 inhibits glioblastoma progression and its downregulated expression indicates a poor prognosis.

## 1. Introduction

Glioma is the most common type of adult brain malignancies, which can be divided into astrocytoma, oligodendroglioma, oligoastrocytoma, and glioblastoma (GBM) according to the histological characteristics. Among them, GBM is the most malignant subtype and is also named as WHO grade IV glioma. Despite comprehensive treatment, the median overall survival time of GBM is only 12–16 months, which is far from satisfactory [1]. Therefore, detailed disease progression mechanisms and crucial biomarkers are essential for prognosis prediction and therapy development.

NKD inhibitor of Wnt signaling pathway 1 (NKD1) was firstly identified in 2001 in both mouse and human, which has 10 exons and is located in chromosome 16q12 of humans [2, 3]. NKD1 is reported to act as an antagonist of both the canonical and noncanonical Wnt-beta-catenin signaling pathways [4], thus participate in chicken embryonic development [5] although another study reported NDKs were dispensable for mice embryonic development [6].

As a negative feedback regulator of the Wnt signaling pathway, NKD1 is initially recognized with anticancer potentials. Mutation of NKD1 may result in a deficient at inhibiting Wnt signaling due to its disability to bind and destabilize Dishevelled (Dvl) proteins [7]. Abnormal expression of NKD1 has also been identified in many malignancies. For example, low NKD1 enhances invasive capacity of NSCLC and correlates with unfavorable prognosis [8], while hypomethylation and high expression of NKD1 indicates better survival [9]. Downregulation of NKD1 was also correlated with a worse prognosis of breast invasive ductal carcinoma [10]. Consistent data were observed in acute myeloid leukemia, osteosarcoma, and uterine corpus endometrial carcinoma [11–13].

Nevertheless, the expression of NKD1 seems distinct in some other malignancies. For example, Arend Koch and his colleagues observed an elevated level of NKD1 in hepatoblastoma [14] although low NKD1 expression was observed in hepatocellular carcinoma [15]. Similarly, NKD1 overexpression was identified in papillary predominant adenocarcinoma [16]. Furthermore, NKD1 was elevated in specific mouse models of intestinal tumors comparing to healthy tissues, which represents a biomarker of tumor growth [17]. Moreover, NKD1 is highly expressed in colon carcinomas and enhances colon cancer growth according to both bioinformatics analyses and experimental validations [18].

Therefore, NKD1 shows distinct expression patterns in different tumor types. However, its expression and function in brain tumors remain unknown. Here, we tested the protein expression of NKD1 in GBMs for the first time and revealed its clinical significance in predicting GBM prognosis. Moreover, we conducted bioinformatics analyses and cellular experiments to validate its tumor-related roles in GBM.

## 2. Methods

### 2.1. Patients and Samples

We retrospectively enrolled 66 adult GBM patients who underwent surgical intervention in our hospital. All the specimens were confirmed as primary GBM according to pathological test. After exclusion, none of the patients had distant metastasis or previous malignant history at the time of diagnosis. In addition, patients who survived less than one month had been excluded. The median age of enrolled patients was 35 years old, ranging from 18–80 years old. The median follow-up time was 47.5 months, ranging from 1–93 months.

### 2.2. Online Datasets

The mRNA expression level of NKD1 was retrieved from TCGA (https://portal.gdc.cancer.gov/) and GTEx (https://gtexportal.org) datasets [19]. The mRNA levels were inverted into FPKM (fragments per kilobase) or TPM (transcripts per million). The immune cell enrichment information was retrieved from the data by Bindea et al. [20]. Data were analyzed via GEPIA online server (https://gepia.cancer-pku.cn/) and compared by the Person chi-square test or Spearman correlation test.

### 2.3. Immunohistochemistry (IHC) Staining

IHC staining was performed to evaluate the protein expression level of NKD1 in GBM tissues. Formalin-fixedparaffin-embedded specimens were cut into 4 *μ*m slides, deparaffinized and hydrated. Then, slides underwent epitope retrieval using the heat-induced method in 90°C water for 1 hour. Then, slides were treated with peroxidase to block endogenous reactions. Specific anti-NKD1 primary antibody (1: 100 dilution) was used to incubate with mentioned slides overnight at 4°C. The antibody used for IHC staining was rabbit polyclonal NKD1 antibody (ab185082, Abcam). On the next day, slides were subsequently incubated with HRP (horseradish peroxidase)-linked secondary antibody and then underwent diaminobenzidine staining, followed by finally counterstained with Hematoxylin. IHC images were independently assessed by two pathologists to distinguish the high-NKD1 expression or low-NKD1 expression of each specimen. The final expression group was discussed by the two pathologists once there existed divergence.

### 2.4. Cell Culture and Transfection

Two human GBM originated cell lines, U87 and U251, were purchased from ATCC. Cells were cultured in DMEM medium supplied with 10% fetal bovine serum (FBS) and 1% penicillin/streptomycin. All cells were cultured at 37°C in a humidified atmosphere containing 5% CO2. Cells were transiently transfected with pcDNA3-NKD1 plasmids (YSY Biotech, Nanjing, China) using pcDNA3-vector as negative control [18] by Lipo3000 reagent (Thermo Fisher Scientifics, Pittsburgh, USA).

### 2.5. Quantitative Reverse Transcription PCR (RT-qPCR)

RT-qPCR was conducted as previously described to measure the mRNA levels. Briefly, transfected cells were lysed and cDNA was extracted. The extracted cDNA was subjected to quantitative reverse transcription according to the manufacturer's instructions. The primers for PCR analyses were as follows: NKD1 forward, 5′-TCGCCGGGATAGAAAACTACA-3′, reverse, 5′-CAGTTCTGACTTCTGGGCCAC-3′;*β*-actin forward, 5′-ATAGCACAGCCTGGATAGCAACGTAC-3′, reverse, 5′-CACCTTCTACAATGAGCTGCGTGTG-3′ [8].

### 2.6. Colony Formation

Transfected cells were seeded into 6-well plates at a density of 250 cells/well and cultured for 10 days at 37°C in a humidified atmosphere containing 5% CO2. During the culturing, the medium was replaced every 4 days. After 10 days, formed colonies were fixed with methanol for 10 min, followed by crystal violet staining for another 15 min. The colony numbers were counted and recorded.

### 2.7. MTT (3-(4,5-Dimethylthiazol-2-yl)-2,5-Diphenyltetrazolium Bromide) Assay

MTT strategy was introduced to evaluate the cell proliferation curve. Briefly, transfected cells were inoculated into a 96-well plate at 1000 cells/well in 100 *μ*l volume. After culturing for 4 hours to allow cell adhesion, cells were further cultured for different designated time points. At each time point, 10 *μ*l of MTT solution was added into each well and incubate for 3 hours in the incubator. Afterwards, the medium were removed and MTT crystals were solved. Finally, the absorbance was recorded at 550 nm using a microplate reader. The experiment was conducted in triplicate and repeated three independent times.

### 2.8. Statistics

Cancer-specific survival was defined as the time period from disease diagnosis to the date of GBM-related death or the date of last follow-up. Survival analysis was performed using the univariate and multivariate Cox hazard regression method. SPSS 22.0 and GraphPad Prism 7.0 Software were used for data analyses [21]. *P* < 0.05 was defined as statistical significance. NS indicates no significance, ^*∗*^indicates *P* < 0.05, ^*∗∗*^indicates *P* < 0.01, and ^*∗∗∗*^indicates *P* < 0.001.

### 2.9. Ethics

This study was approved by the Affiliated Hospital of Inner Mongolia Medical University Ethic Committee. Written informed consent was obtained from each participant.

## 3. Results

### 3.1. NKD1-mRNA Level Is Higher in GBMs than Normal Brains or Other Glioma Subtypes

**Figure 1:**
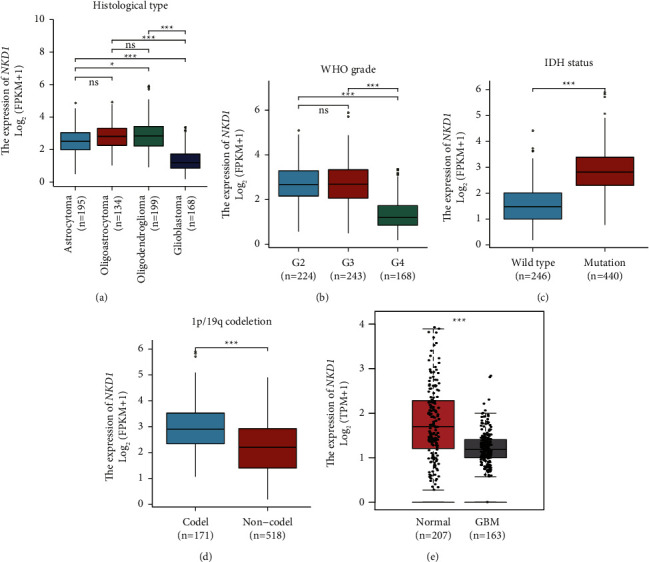
Expression difference of NKD1-mRNA in different glioma subtypes: (a) comparison of NKD1-mRNA level in different glioma histological subtypes, (b) comparison of NKD1-mRNA level in gliomas with different WHO grades, (c) comparison of NKD1-mRNA level in gliomas with wild type or mutated IDH, (d) comparison of NKD1-mRNA level in gliomas with 1p/19q codeletion or non-codeletion, and (e) comparison of NKD1-mRNA level in normal brain tissues and glioblastomas.

We firstly analyzed the mRNA level of NKD1 in glioma tissues from the TCGA dataset. Accordingly, astrocytoma, oligoastrocytoma, and oligodendroglioma showed similar NKD1-mRNA level without statistically significant difference. However, GBM tissues contain significantly lower NKD1-mRNA level than the other three histological types previously (Figure 1(a), *P* < 0.001). Consistently, by dividing patients based on the WHO grade, we found that Grade IV GBM showed significantly a lower NKD1-mRNA level than Grade II or Grade III gliomas (Figure 1(b), *P* < 0.001). Interestingly, the NKD1-mRNA level was significantly lower in gliomas with wild type IDH than those with mutated IDH (Figure 1(c), *P* < 0.001). This was consistent with the previous findings since most primary GBMs showed wild type IDH (Isocitrate dehydrogenase 1), while low-grade gliomas showed a higher mutated IDH rate [22]. It has been well-recognized that gliomas with 1p/19q codeletion possess better prognosis than those with noncodeletion [23], therefore, we next compared whether NKD1-mRNA show any expression difference in those two types. As a result, NKD1-mRNA level was significantly higher in 1p/19q codeletion specimens (Figure 1(d), *P* < 0.001), suggesting that high NKD1 may help predict a better prognosis. However, the TCGA dataset contains limited normal brain tissue samples; therefore, we also retrieved NKD1-mRNA information from the GTEx dataset to compare the difference between GBMs and normal brain tissues. As shown in Figure 1(e), NKD1-mRNA level was significantly downregulated in GBMs compared to that in normal brains (*P* < 0.001).

**Figure 2:**
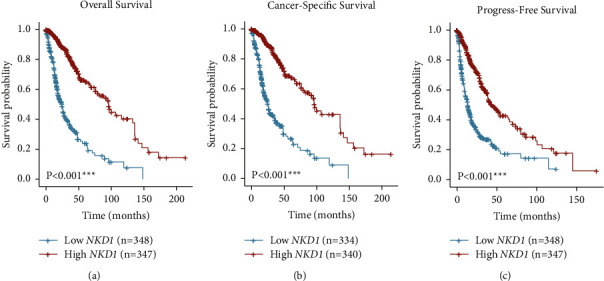
Survival analyses of glioma patients based on the mRNA level of NKD1. The survival analyses were conducted using the Kaplan–Meier method according to the TCGA dataset, which reflected that higher-NKD1 mRNA level was correlated with better overall survival (a), cancer-specific survival (b), and progress-free survival (c) of glioma patients. Data were compared by log-rank test.

Next, we analyzed whether NKD1 level has any prognostic significance for glioma prognosis (Figures 2(a)–2(c)). As expected, low NKD1 is significantly correlated with worse overall survival, cancer-specific survival, and progress-free survival of glioma (all *P* < 0.001). In other words, lower-NKD1 mRNA level may predict poor glioma prognosis. Therefore, we were engaged to further investigate the prognostic role of NKD1-protein level in another retrospective cohort from our medical center.

### 3.2. Patients' Information

Among the 66 enrolled surgical-treated GBM patients in our medical center, there were 29 females and 37 males. The entire diagnostic age was young with a median age of 35 years old, 36 cases were younger than 40 years old, while the other 30 cases were older. Among them, 6 patients showed tumor location in the parietal lobe, 24 cases in the temporal lobe, 32 cases in the frontal lobe, and the other 4 cases with unclear description about the detailed tumor location. The median tumor size is 2.4 cm in diameter, ranging from 0.8–7.5 cm. According to personalized disease status, 37 cases underwent local resection, 12 cases underwent radical resection, while the other 17 cases underwent lobectomy. Till the end of follow-up, 18 cases were identified as disease-specific survival.

### 3.3. NKD1 Serves as a Novel Prognostic Factor for GBM

**Figure 3:**
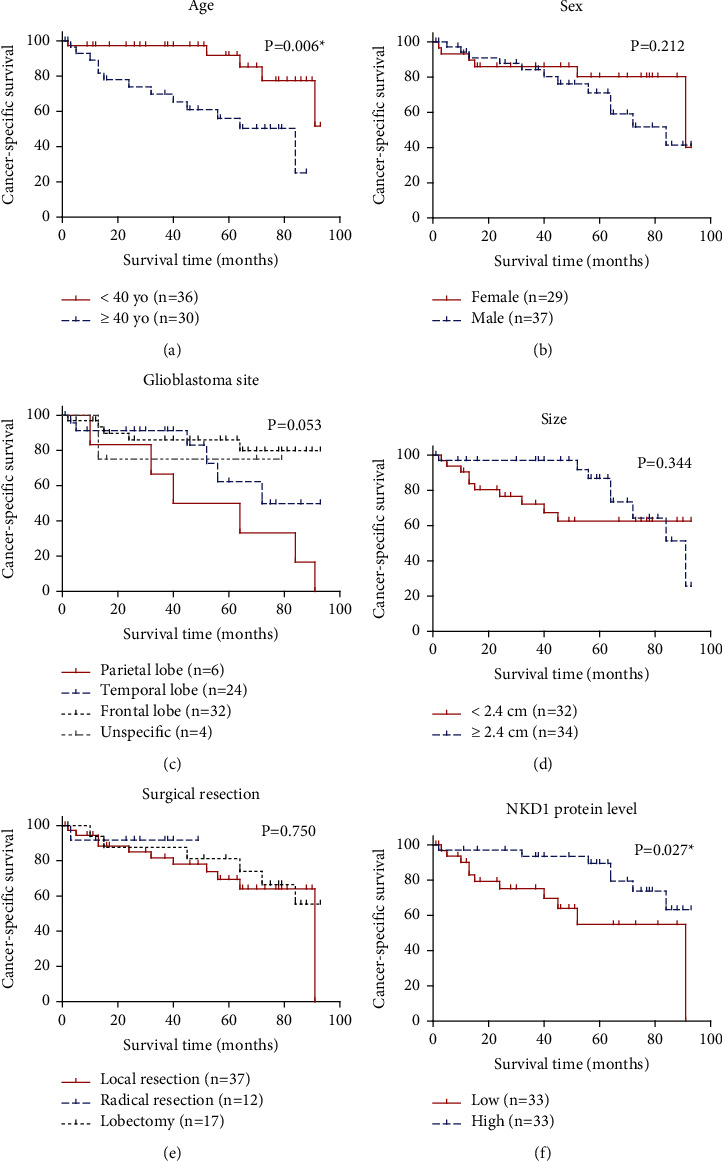
Survival analyses of GBM patients in our medical center. Cancer-specific survival analyses were conducted according to patients' age (a), sex (b), glioblastoma site (c), tumor size (d), surgical resection pattern (e), and NKD1 protein level (f), respectively.

We next conducted survival analyses based on each clinical variable (Figure 3). As expected, elder patients exhibited worse prognosis than the younger ones (Figure 3(a), *P* = 0.006), while females and males showed no significant difference in cancer-specific survival (Figure 3(b), *P* = 0.212). Although patients with parietal lobe tumor location seemed to had worse prognosis, the difference was not statistically significant (Figure 3(c), *P* = 0.053). Surprisingly, neither tumor size (Figure 3(d), *P* = 0.344) nor surgical pattern (Figure 3(e), *P* = 0.750) showed a significant effect on patients' survival perhaps due to limited case numbers.

As described in the method section, all the collected GBM tissue samples were subjected to IHC analyses to subgroup patients into low-NKD1 protein expression group and high-NKD1 protein expression group (Supplemental Figures S1A and S1B). Accordingly, 33 patients were characterized with low-NKD1 protein level, while the other 33 cases with high-NKD1 protein level. Survival analysis revealed that patients with low NKD1 protein levels in GBM samples exhibited significantly worse cancer-specific survival than those with high-NKD1 protein levels (Figure 3(f), *P* = 0.027).

The multivariate Cox regression model was further used to identify independent prognostic factors (Table 1). As a result, elder age was identified as an independent unfavorable factor (HR = 6.0, 95% CI 1.9–19.2, and *P* = 0.003), while frontal lobe tumor location was identified as an independent favorable factor (HR = 0.2, 95% CI 0.1–0.7, and *P* = 0.011). Of note, a higher NKD1 expression level was also confirmed as an independent favorable prognostic factor of GBM for the first time (HR = 0.3, 95% CI 0.1–0.8, and *P* = 0.019).

### 3.4. NKD1 Inhibits GBM Growth and Shows Cross-Talk with T Cell Infiltration

**Figure 4:**
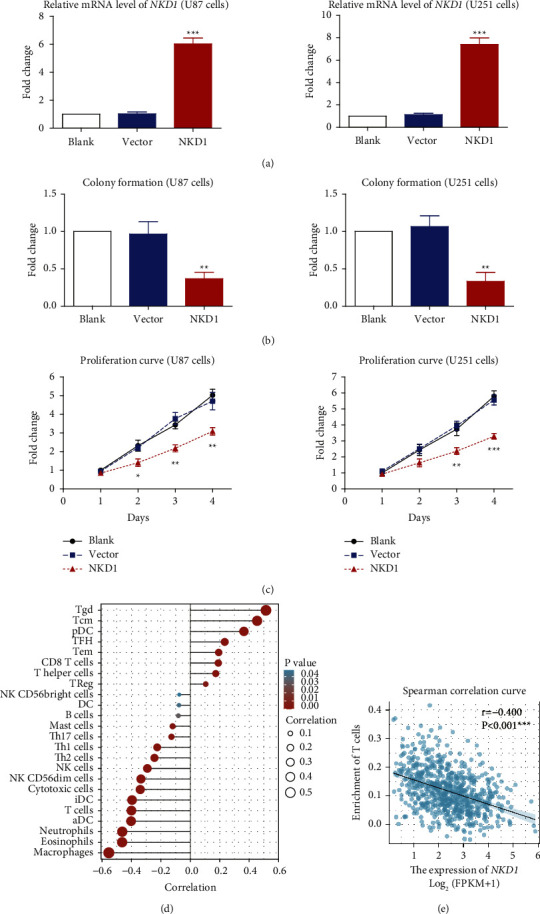
NKD1 is correlated with immune cell enrichment and inhibits GBM growth. (a) RT-qPCR method was used to validate transfection efficiencies of pcDNA3-NKD1 and pcDNA3-vector plasmids. The blank group referred to cells treated with only transfection reagent without any plasmid. (b) Colony formation assay was conducted to evaluate GBM cell proliferation capacity. (c) The MTT method was used to dynamically monitor the growth curves of transfected GBM cells. (d) The correlations between immune cell enrichment and NKD1-mRNA level in GBM tissues were summarized. (e) Spearman correlation test indicated a negative correlation between NKD1-mRNA level and T cell infiltration in GBM.

Since clinical evidence implied a potential tumor-suppressing role of NKD1, we next conducted cellular experiments to validate its detailed effects in GBM. NKD1 plasmids were transfected into U87 and U251 cells, respectively. RT-qPCR data confirmed the transfection efficiencies compared to blank control and vector control (Figure 4(a)). Both the colony formation assay and MTT proliferation assay revealed an attenuated GBM growth after overexpressing NKD1 (Figures 4(b) and 4(c)), highlighting the crucial role of NKD1 as a novel tumor suppressor.

In addition, we analyzed the correlation between NKD1 level and immune cell enrichment (Figure 4(d)), which showed a negative correlation with T cells, neutrophils, and macrophages. For example, a lower mRNA level of NKD1 was significantly correlated with upregulated T cell infiltration in GBM (Figure 4(e), *P* < 0.001), indicating the potential role of NKD1 in the immune environment during GBM progression.

## 4. Discussions

For the first time, our data revealed a downregulated expression level of NKD1 in GBM on both mRNA and protein levels. RNA transcription and subsequent protein expression are negatively modulated by upstream methylation. Consistently, high methylation of NKD1 CpG island is significantly correlated with a worse prognosis of epithelial ovarian cancer, which is independent from other clinical parameters according to multivariate Cox model analysis [24]. Similarly, a later study defined NKD1 methylation as an important unfavorable prognostic factor for a risk model of high-grade serous ovarian cancer [25]. NKD1 promoter was reported to be hypermethylated in U87 cell lines, however, its hypermethylation was not identified in any gliomas (*n* = 70) according to Gotze's et al. data [26]. In contrast, NKD2-hypermethylation occurred in 43% (13/30) of the primary glioblastoma tissues, while a super low rate was observed in astrocytoma (1/30).

Besides methylation, we should also keep in mind that post-translational protein modifications are critical for protein functions [27]. For example, the myristoylation of NKD2 is important for its plasma membrane localization since its myristoylation-deficient mutant is only localized in cytoplasmic. Moreover, myristoylation of NKD2 antagonizes the Wnt-beta-catenin signaling pathway by degrading membrane-localized Dvl-1 [28]. Interestingly, Koch et al. reported that NKD1 in hepatoblastomas with betacatenin mutations had no antagonistic effect [14], which may result from specific modifications but require further investigations.

Clinical data indicated that low NKD1 level was significantly correlated with unfavorable GBM prognosis by both univariate and multivariate analyses. Moreover, we initially provided evidence that overexpressing NKD1 can significantly suppress the proliferation of GBM cells. However, our study has several limitations. Firstly, we did not fully dig into the functional mechanisms of NKD1 in inhibiting GBM progression. Previous studies indicated that NKD1 interacts with Axin [29] and prevents nuclear accumulation of *β*-catenin [30], subsequently suppress Wnt signaling; whether NKD1 inhibits GBM growth through these mechanisms need detailed illuminations. Meanwhile, our data suggested that NKD1 expression was negatively correlated with T cell infiltration; therefore, NKD1 may also be involved in the immunological microenvironment during GMB development. Secondly, our medical center has a limited GBM case number and we only enrolled 66 cases in our retrospective cohort to test NKD1 protein expression level. Therefore, further evidence is necessary from more cases. Anyway, we believe our major conclusion because lower NKD1-mRNA level also indicates worse GBM prognosis in TCGA datasets, which is consistent with its protein significance in our cohort. Thirdly, recent studies suggested that NKD1 could serve as an independent predicting biomarker for tumor responsiveness of neoadjuvant chemo-radiotherapy in rectal cancer [31, 32], and whether NKD1 can help direct chemotherapy of gliomas deserve further investigations.

## 5. Conclusions

NKD1 shows a decreased expression level in GBMs compared to normal brains or other glioma types, and its low expression results in poor GBM prognosis via enhancing GBM progression.

## Figures and Tables

**Table 1:** Cancer-specific survival analyses.

Variables	Cases (*n* = 66)	Univariate analysis		Multivariate analysis	
		HR (95% CI)	*P* value	HR (95% CI)	*P* value
Age					
<40 yo	36	Reference		Reference	
≥40 yo	30	4.8 (1.6–15.0)	0.006^*∗∗*^	6.0 (1.9–19.2)	0.003^*∗∗*^
Sex					
Female	29	Reference			
Male	37	1.8 (0.7–4.9)	0.212		
GBM site					
Parietal lobe	6	Reference		Reference	
Temporal lobe	24	0.4 (0.1–1.2)	0.094	0.6 (0.2–2.0)	0.403
Frontal lobe	32	0.2 (0.1–0.6)	0.006^*∗∗*^	0.2 (0.1–0.7)	0.011^*∗*^
Unspecific	4	0.3 (0.04–2.8)	0.316	0.4 (0.1–3.7)	0.430
GBM size					
<2.4 cm	32	Reference			
≥2.4 cm	34	0.6 (0.3–1.6)	0.344		
Surgery					
Local resection	37	Reference			
Radical resection	12	0.5 (0.1–4.2)	0.540		
Lobectomy	17	0.8 (0.3–2.1)	0.600		
NKD1 expression					
Low	33	Reference		Reference	
High	33	0.3 (0.1–0.9)	0.027^*∗*^	0.3 (0.1–0.8)	0.019^*∗*^

HR: hazard ratio, GBM: glioblastoma, and NKD1: NKD inhibitor of Wnt signaling pathway 1.

## Data Availability

Data used to support the findings of this study are available upon request.

## Supplementary Materials

Supplementary Materials Supplementary Figure S1: IHC staining results of NKD1 protein in GBM tissues: (A) representative low NKD1 protein expression in GBM as reflected by IHC and (B) representative high-NKD1 protein expression in GBM tissues.
