# Supplementary material for: Clinical Significance of NKD Inhibitor of WNT Signaling Pathway 1 (NKD1) in Glioblastoma
Source: Genet Res (Camb). 2023 Mar 17;2023:1184101. doi: 10.1155/2023/1184101 (PMC10038739; doi:10.1155/2023/1184101)

**Figure S1. IHC staining results of NKD1 protein in GBM tissues.**

(A) Representative low NKD1 protein expression in GBM as reflected by IHC.

(B) Representative high NKD1 protein expression in GBM tissues.


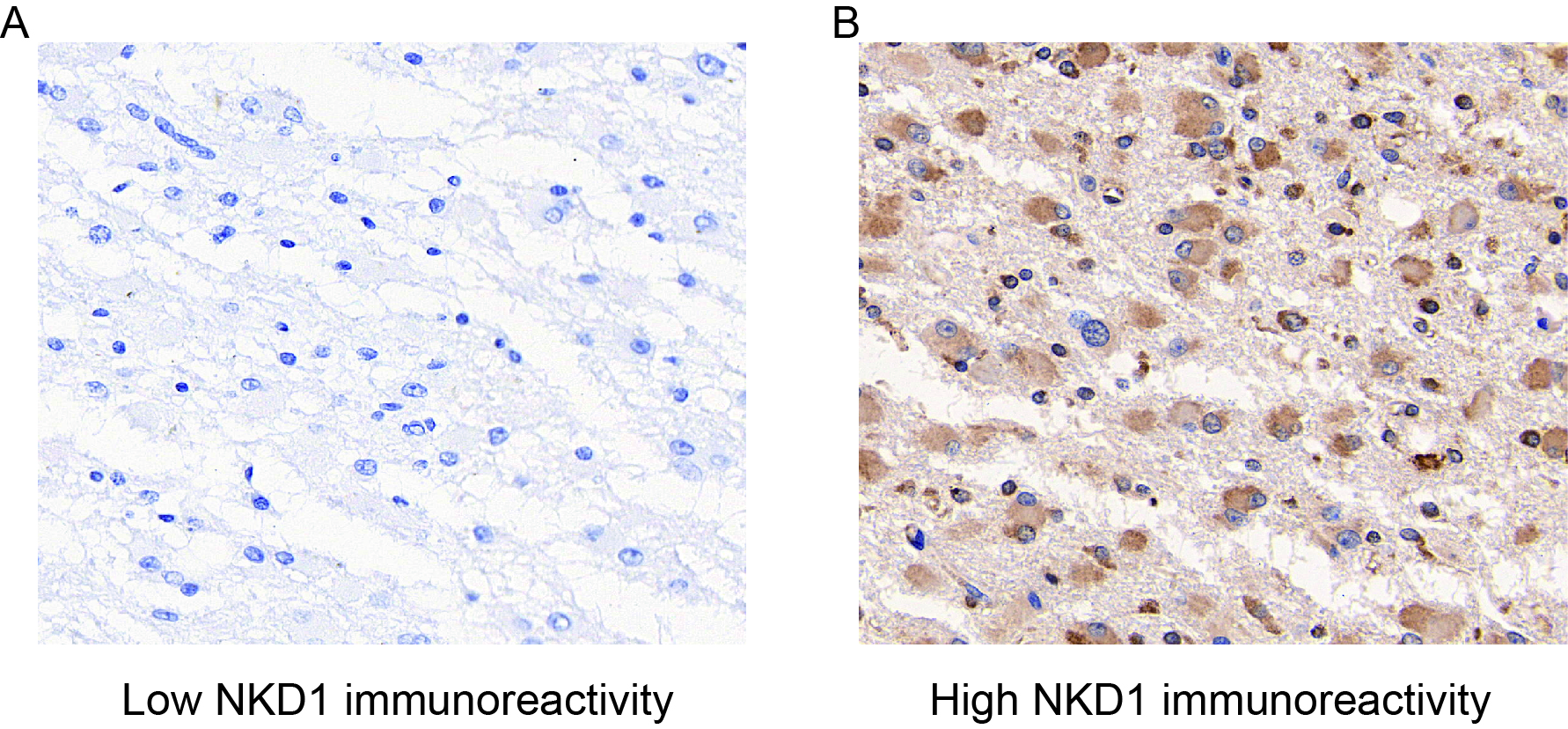

Supplement: Supplementary Materials — Supplementary Figure S1: IHC staining results of NKD1 protein in GBM tissues: (A) representative low NKD1 protein expression in GBM as reflected by IHC and (B) representative high-NKD1 protein expression in GBM tissues. [file 1184101.f1.zip › Supplemental file.docx]
